# Metformin affects thyroid function in male rats

**DOI:** 10.18632/oncotarget.22536

**Published:** 2017-11-20

**Authors:** Xiaowen Hu, Yang Liu, Changmei Wang, Lulu Hou, Xiaoyan Zheng, Yeqiu Xu, Lin Ding, Shuguang Pang

**Affiliations:** ^1^ Department of Infectious Diseases, Jinan Central Hospital Affiliated to Shandong University, Jinan 250013, China; ^2^ Department of Endocrinology, Jinan Central Hospital Affiliated to Shandong University, Jinan 250013, China; ^3^ School of Chemistry and Molecular Biosciences, The University of Queensland, St Lucia Qld 4072, Australia

**Keywords:** metformin, thyrotropin, free triiodothyronine, free thyroxine, type 2 diabetes

## Abstract

An intriguing area of research in type 2 diabetes recently discovered association of metformin therapy with thyroid functional and morphological changes. We aimed to evaluate the external symptoms and biochemical indicators concerning thyroid function in rats treated with metformin. Male wistar rats were randomly divided into four groups: Group (D–/M–), Group (D–/M+), Group (D+/M–), and Group (D+/M+), according to whether they were induced to diabetic model or placed on metformin. Characteristics of food intake, body weight, and other external symptoms were recorded. Thyroid function, concluding serum thyrotropin (TSH), free triiodothyronine (FT3), free thyroxine (FT4), were measured. We found a significantly higher TSH and lower FT4 in rats in Group (D+/M–), compared with rats in Group (D–/M–), but no significant change in FT3 level. Rats on metformin treatment exhibited relatively lower body weight and symptoms like irritability and diarrhea, concomitant with marked increase in FT3 and FT4 , no matter if they were induced to diabetic model or not . A slight but significant reduction in TSH concentration was also observed in rats received metformin. These data reveal that metformin can modify thyroid function with corresponding clinical symptoms of hyperthyroidism in male rats. Metformin's contribution to suppress TSH and increase FT3, FT4 should arise our attention to its treatment interference in clinical practice.

## INTRODUCTION

Diabetes mellitus and thyroid disorders are the two most common endocrinopathies. Both conditions frequently coexist and the prevalence of thyroid dysfunction in diabetic patients is higher than that in the general population [1, 2]. A large scale study observed a prevalence of thyroid dysfunction of 13.4% in 1301 clinic patients with diabetes while the prevalence of thyroid dysfunction has been reported as 6.6% in the background population [3].

Metformin, a commonly used anti-diabetic drug over the years, is currently recommended for prevention or delay of type 2 diabetes (T2D), besides as first-line therapy for T2D. Its main effect is to ameliorate hyperglycemia by reducing hepatic glucose production, improving hepatic insulin resistance and enhancing glucose uptake and utilization by peripheral tissues [4]. Metformin also exerts multiple further properties recent years, for instance, it is used in polycystic ovarian syndrome (PCOS) and anti-proliferative activity [5, 6].

Interestingly, some studies showed that metformin would effect the levels of thyrotropin (TSH) without relevant changes in serum thyroxine (T4) and triiodothyronine (T3) levels or clinical symptoms of hyperthyroidism. And these findings have been evaluated in different clinical settings (patients receiving metformin for diabetes [7, 8] or PCOS [9, 10], with or without thyroid dysfunction [11–13]). In general, clinical studies supported the TSH inhibition of metformin in patients with diabetes or PCOS, even together with L-thyroxine (L-T4) alternative therapy. Recently, clinical trials also indicated that metformin therapy can decrease thyroid volume and nodule size in persons with insulin resistance [14]. Still, metformin may inhibit the growth, migration, and mesenchymal transition of thyroid cancer [15, 16]. But the conclusion can not be completely consistent. In a cross-sectional study, Diez *et al*. found that the TSH levels were higher in metformin treated group than metformin untreated [17]. And an opposite finding indicated neither use of metformin nor of any other anti-diabetic drug was associated with a decreased risk of thyroid cancer [18]. Therefore, it is necessary to further investigate the thyroidal effect of metformin and it has important clinical implications for treatment requirements.

At present, metformin on thyroid functions is limited to clinical research. The effect of metformin on thyroid hormone changes, morphology and its mechanism are still lack of further animal experimental study. We constructed diabetic rat models and treated with metformin . We divided the rats into four groups, Group (D–/M–), Group (D–/M+), Group (D+/M–), and Group (D+/M+) respectively. We recorded the changes that the drug affects FT3, FT4 and TSH in all the rats. We hope to clarify the effect of metformin on thyroid hormones to guide the clinical application in the treatment for diabetic patients and/or with thyroid dysfunction .

## RESULTS

### The general features of diabetic rats and the effects of metformin on glucose, insulin

**Figure 1:**
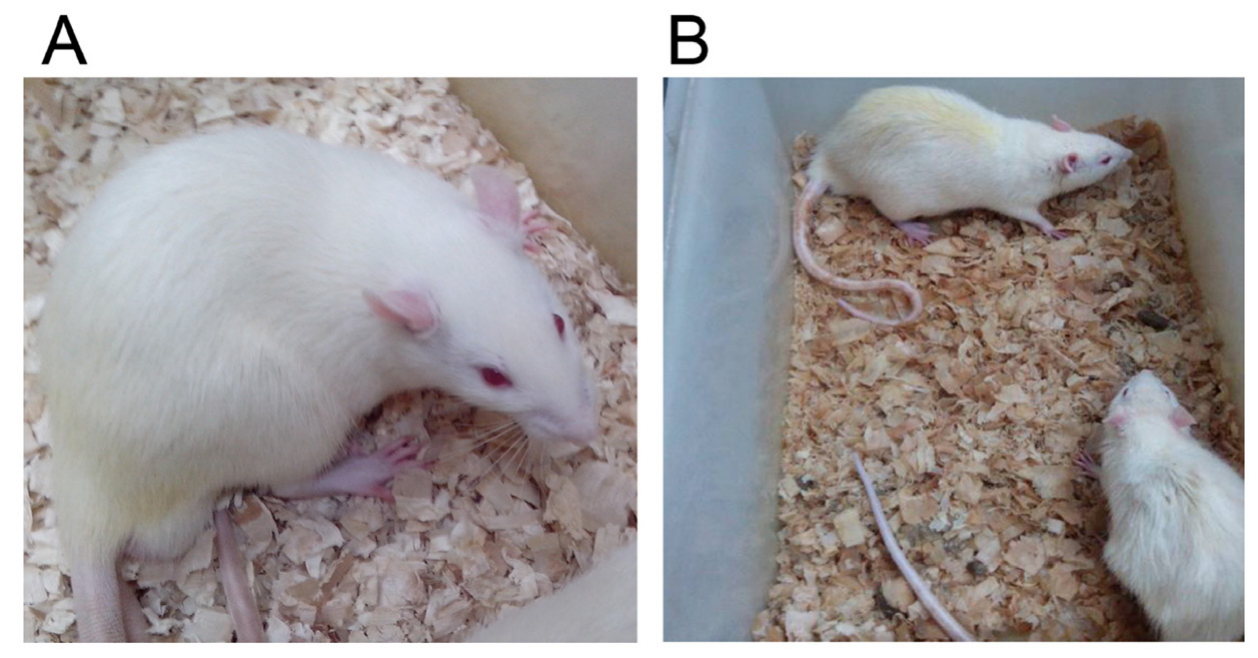
The general features of rats in Group (D–/M–) and Group (D+/M–) Diabetic model (**B**) presented dim and disorderly hair, polyuria (dirty and humid underpad), weight loss and sluggishness, as against the normal control group (**A**).

**Figure 2:**
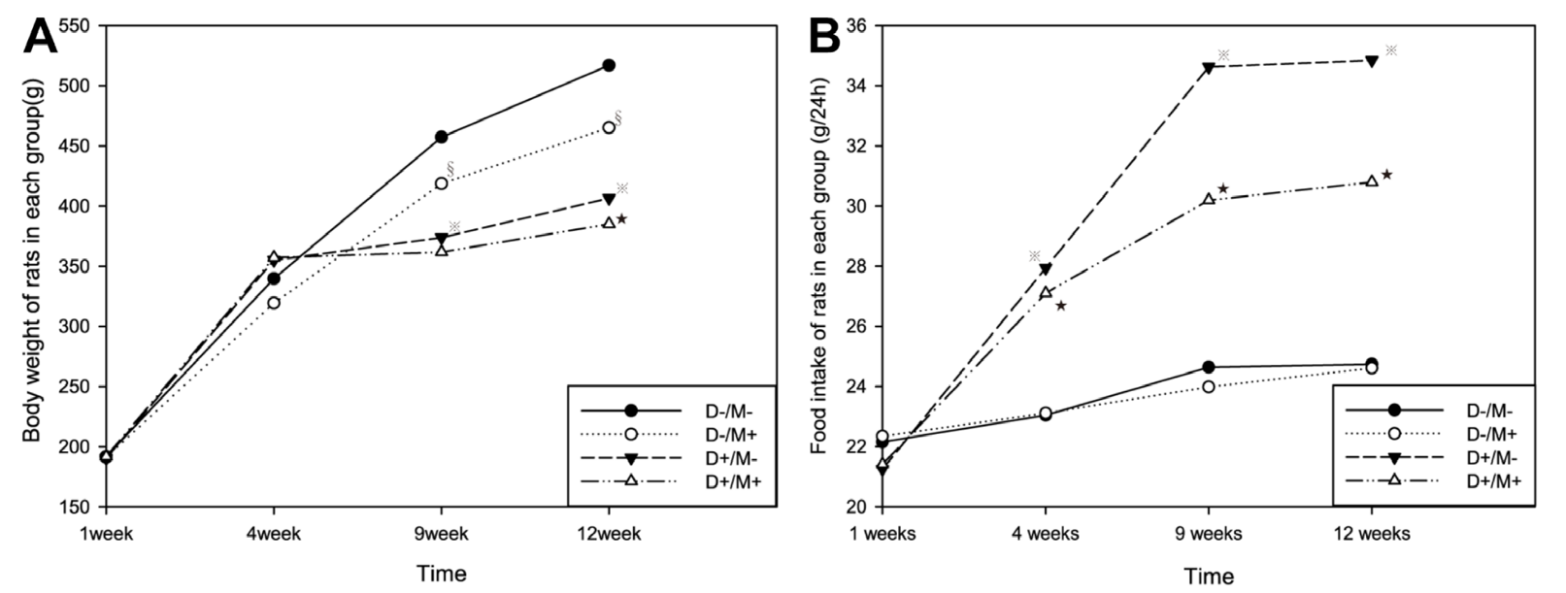
Changes in body weight and food intake (**A**) The body weight values of all the rats in each group every week. (**B**) The weekly food intake of all the rats. Diabetic rats showed weight loss and increased food intake compared with non-diabetic rats. Rats received metformin showed relatively less body weight . (^※^*p* < 0.05 vs. Group (D–/M–), ^★^*p* < 0.05 vs. Group (D–/M+), ^§^*p* < 0.05 vs. Group (D–/M–)). Data were presented as mean ± SD. Comparison of two group means was made with the *t*-test, a difference of *p* < 0.05 was considered statistically significant.

**Figure 3:**
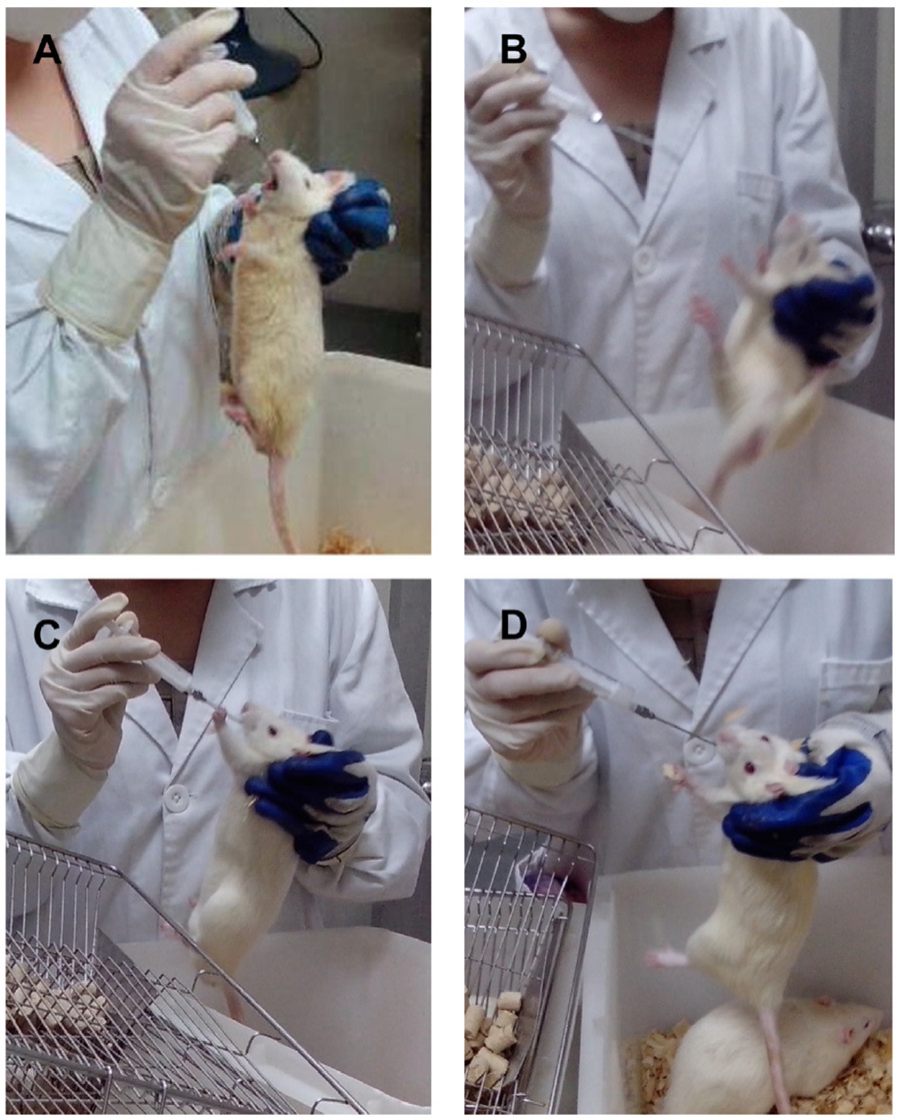
Symptoms of hyperthyroidism of rats on metformin Compared with rats in group without metformin (**A**), rats in Group (D–/M+) and Group (D+/M+) exhibited symptoms like irritability, hyperactive movement and bad compliance with intragastric administration (**B**, **C**, **D**).

Rats in Group (D+/M–) and Group (D+/M+) showed hyperphagia, polydipsia, polyuria and weight loss after STZ induction (Figure 1). The increase in plasma glucose reached a plateau within 3 days and the plasma glucose varies from 17.9 to 26.8 mmol/L on the 72 nd hour after injection of STZ. Among the 20 rats receiving STZ, 19 rats got stable blood glucose over 16.7 mmol/L and were taken as successful diabetic model. One died as a result of high plasma glucose. The FPG, FINS and HOMA-IR were examined at the end of the experiment (Table 1). Diabetic rats (D+/M–) had almost 3 fold higher of FPG and 2 fold higher of FINS than that in control rats (D–/M–) (All *p* < 0.05). According to HOMA-IR index, Diabetic rats showed significant insulin resistance. As expected, food intake and body weight increased gradually before injection of STZ. Compared with control groups, rats received metformin showed relatively small amount of food intake and body weight (Figure 2), except for symptoms like irritability and diarrhea. Irritability means that all the rats received metformin presented hyperactive movement and bad compliance on intragastric administration than rats without metformin treatment (Figure 3).

**Table 1:** FPG, FINS and HOMA-IR in each group

Group	*N*	FPG (mmol/L)	FINS (mIU/L)	HOMA-IR
D–/M–	10	5.30 ± 0.58	21.92 ± 2.72	5.15 ± 0.73
D–/M+	10	4.94 ± 0.99	23.42 ± 2.35	5.17 ± 1.24
D+/M–	10	19.20 ± 2.04^*^	50.95 ± 9.67^*^	43.72 ± 10.49^*^
D+/M+	9	17.34 ± 2.37	48.70 ± 9.75	37.79 ± 10.23^#^

Data was expressed as mean SD.

^*^p < 0.05, compared with Group (D–/M–).

^#^*p* < 0.05, compared with Group (D+/M–).

### Changes of thyroid function in diabetic rats and the effects of metformin on serum TSH, FT3 and FT4 levels

**Figure 4:**
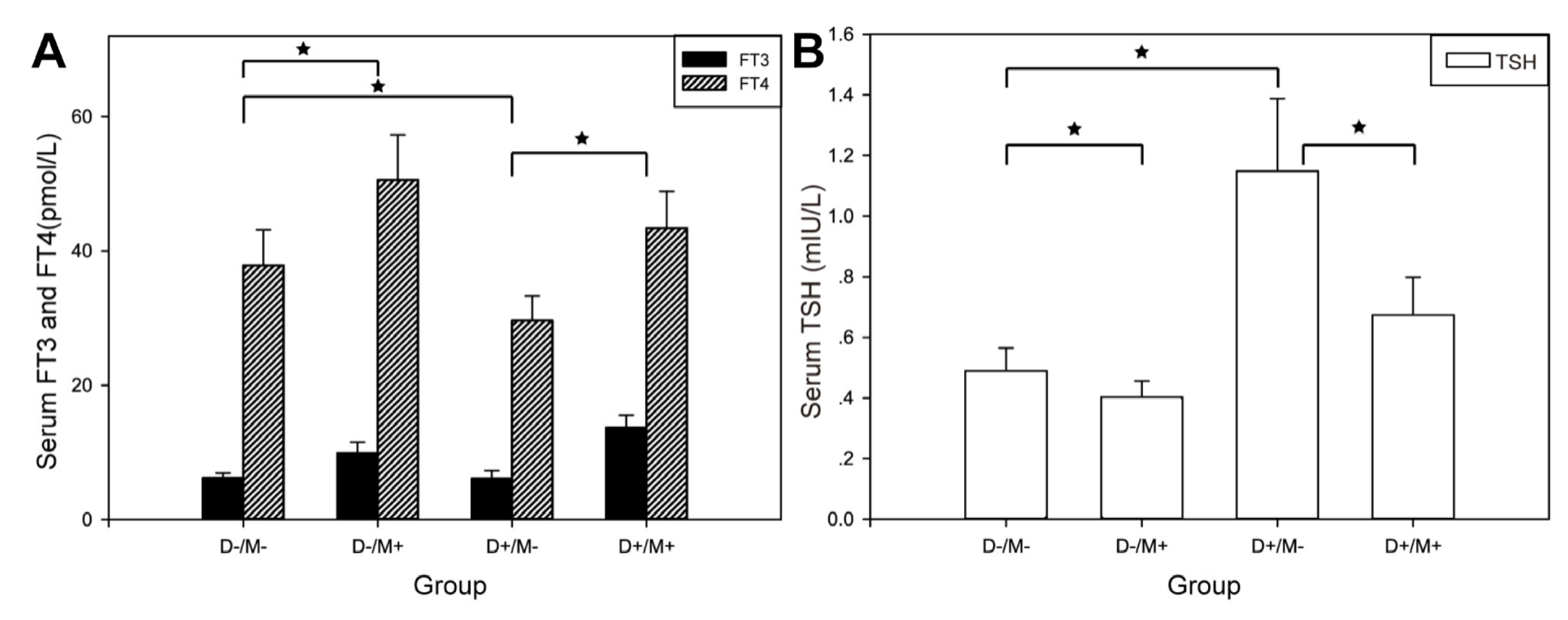
Thyroid function in each group (**A**) Comparison of FT3, FT4 of each group. (**B**) Comparison of TSH of each group. SD was represented by the bar above the histogram, and data between two groups with statistical significance were marked by^★^.

TSH in Group (D+/M–) rats (1.15 ± 0.05 mIU/l) was significantly higher than that in Group (D–/M–) rats (0.49 ± 0.08 mIU/l). FT4 in Group (D+/M–) rats (37.86 ± 5.27 mIU/l ) reduced signifcantly compared to Group (D–/M–) rats (29.64 ± 3.66 pmol/l), but there was no significant change in FT3 (6.23 ± 0.72 vs. 6.15 ± 1.15 pmol/l) level. Rats on metformin treatment exhibited marked increase in FT3 and FT4 significantly, no matter if they were induced to diabetic model. A slight but significant reduction in TSH concentrations was observed in rats received metformin (Figure 4, Table 2).

**Table 2:** TSH, FT3 and FT4 in each group

Group	*N*	TSH (mIU/l)	FT3 (pmol/l)	FT4 (pmol/l)
D–/M–	10	0.49 ± 0.08	6.23 ± 0.72	37.86 ± 5.27
D–/M+	10	0.40 ± 0.05^*^	9.93 ± 1.63^§^	50.55 ± 6.76^§^
D+/M–	10	1.15 ± 0.05^*^	6.15 ± 1.15	29.64 ± 3.66^#^
D+/M+	9	0.67 ± 0.14^★^	13.71 ± 1.84^※^	43.39 ± 5.47^※^

Data are expressed as means ± SD.

^*^p < 0.01, versus Group (D–/M–), ^#^*p* < 0.001, versus Group (D–/M–), ^※^*p* < 0.001, versus Group (D+/M–), ^§^*p* < 0.001, versus Group (D–/M–) ,^★^*p* < 0.05, versus Group (D+/M–).

## DISCUSSION

Type 2 diabetes mellitus is a complex disorder characterized by a progressive decline in insulin resistance and followed by the inability of β cells to compensate for insulin resistance. In our study, we constructed T2D rat model according to feed rats with HFD and a low dose of STZ. Researches before have demonstrated that HFD-fed rats with the dose of STZ (30–35 mg/kg) was considered to represent the pathophysiological state of T2D [19, 20]. At the end of the experiment, the rats in group (D+/M–) are in a state of insulin resistance and still have the ability to secrete substantial amount of insulin.

We found a significantly higher TSH and lower FT4 in diabetic rats in Group (D+/M–), compared with normal rats in Group (D–/M–) (*p* < 0.01 for both), but no significant change in FT3 level. This seems to make sense, as another study on rats showed a TSH elevation induced by high-fat diet, which were also given to our diabetic rats [21]. And our results are also in accord with other clinical studies, suggesting that serum FT4 was negatively associated and TSH was positively associated with insulin resistance [22].

Metformin, the most widely used drugs in patients with diabetes, has been reported to induce a reduction in TSH levels in several clinical trials and this reduction effect exists not only in overt and subclinical hypothyroidism, but also in euthyroid patients with higher baseline TSH levels [23, 24]. Mechanism of metformin action on thyroid profile is complex and indefinite. Metformin has recently been shown to cross the blood–brain barrier in the rat experiments [25]. Central effects of metformin on hypothalamic-pituitary-thyroid axis regulation might involve the adenosine monophosphate-activated protein kinase (AMPK) system. Contrary to its peripheral effect, metformin has been proved to inhibit the activity of AMPK in the hypothalamus, which may affect the role of T3 on the hypothalamus, thereby reducing the secretion of TSH [26, 27]. There is also evidence that metformin treatment increases hypothalamic dopaminergic tone in association with improved insulin sensitivity [28]. Metformin can also affect the thyrotropin function in the pituitary gland [29]. Besides, metformin is considered to change the expression and/or affinity of thyroid hormone receptors (TRs) via activation of AMPK in the peripheral tissues [30]. A metformin-induced change in the bioavailability of circulating thyroid hormones appears convincing. Actually, most of the T3 and T4 are combined with thyroxine binding protein in the circulation, whereas the free components of circulating hormones may be bioavailable and correlates with the clinical state of the patient. Increased free thyroid hormones therefore enlarge the negative feedback regulation of TSH.

We observed that rats treated with metformin in our experiments showed symptoms like hyperthyroidism, such as irritability, diarrhoea and weight loss, as well as a decrease in TSH and a marked increase in FT3, FT4 levels, no matter if they were induced to diabetic models or not (*p* < 0.05 for all) and the differences were statistically significant. This is not the same as most of the previous clinical studies that metformin can only reduce the level of TSH in patients. But in one forward-looking study, Isidro *et al*. used metformin to intervene the T2D patients with hypothyroidism. The level of FT4 increased, although the difference was not statistically significant. After three months of discontinuation of metformin, the level of FT4 decreased and the difference was statistically significant [7]. In particular, metformin, has been reported to affect the thyroid sodium-iodide symporter (NIS) protein and iodine uptake. The synthesis of thyroid hormones is associated with a variety of factors and NIS-mediated iodine uptake is the first step [31]. Abdulrahman *et al*. used metformin , as the AMPK agonist, to treat thyroid follicular cells. The iodine uptake and NIS expression were inhibited and this effect could be reversed by compound C, which was the AMPK inhibitor [32]. The studies were consistent with our results, suggesting that metformin may directly affect the synthesis of thyroid hormone and then decrease the level of TSH. Moreover, no study was about the effect of metformin on normal populations so far. For the first time we report that the drug affects thyroid profile in normal rats. Most clinical studies before were retrospective researches and had diverse settings including patients with different indications for metformin treatment (insulin resistance, T2D, or PCOS), or with different thyroid function basis (euthyroidism, overt or subclinical hypothyroidism). Besides, some of the evaluated patients were affected by obesity, which may have an impact on the thyroid hormones [33]. All the factors should be considered to affect the role of metformin on thyroid profiles.

In addition, some limitations in our study need to be noted. We failed to evaluate baseline FT3, FT4 and TSH values and the degree of their changes during metformin treatment. And there is no literature reporting the normal ranges of FT3, FT4 and TSH values of rat either. So it is not sure whether the results we observed are within or outside of the normal ranges. Besides, symptoms such as diarrhea and weight loss are frequently accompany metformin therapy, and may be not specific symptoms of hyperthyroidism. We also failed to record the definite and quantify assessment of hyperthyroidism such as rats heart rate, pulse, blood pressure and so on. Actually, metformin dose used in our animal experiment exceeds conventional dose used for clinical diabetes treatment may account for the results. And only male rats were studied, female animals should be included in further study.

Despite these limitations, the difference of FT3, FT4 and TSH values is meaningful. Metformin-induced thyroid function changes seem to be notable. Ongoing animal research should be useful in guiding design of clinical trials, not only to evaluate metformin at conventional antidiabetic dose, but also to explore more aggressive dosing, which metformin may modify thyroid function . But when TSH is not properly increased, such as the diabetic patients with subclinical hypothyroidism, especially with metabolic diseases or thyroid nodules or even thyroid cancer, the mild but significantly reduced TSH effect of metformin can play a special therapeutic role. Of course, these hypotheses need to be validated in properly designed studies with large scale or a long-term follow-up. Treatment interference, as discussed above for metformin, needs to be taken into account in the course of clinical medication. Also screening for thyroid dysfunction should be recommended as part of routine practice.

## MATERIALS AND METHODS

### Animals

Forty eight-week-old male wistar rats (weight 160–200 g) were obtained from Vital River Laboratory Animal Technology Co. Ltd (Beijing, China). The rats were housed in laboratory animal room of Shandong University (Jinan, China), under standard conditions (constant room temperature of 24 ± 2°C, humidity of 55 ± 5%, laboratory chow and sterile water ad libitum, an alternating 12-hour light/dark cycle). After acclimatization to the environment for one week, the animals were randomly assigned to the following four groups with ten in each group: the control group (D–/M–), the control treated with metformin group (D–/M+), the diabetes group (D+/M–), and the diabetes treated with metformin group (D+/M+), according to whether they were induced to diabetic model or administered with metformin. All animal experiments were performed in accordance with the National Institutes of Health Guide for the Care and Use of Laboratory Animals, and were approved by the ethics committee of Shandong University.

### Diabetic model

Rats in Group (D+/M–) and Group (D+/M+) were induced to type 2 diabetes. Diabetic model was made by high-fat diet throughout the experiment and a low dose intraperitoneal injection of prepared solution of streptozotocin (STZ, Sigma, USA) in citrate buffer (0.1 mol/L, pH 4.5) after an overnight fast at the fifth week. The dose of STZ was 35 mg/kg and therefore the volume was 1 ml/kg. The other rats were fed with standard diet and received an intraperitoneal injection of vehicle solution (citrate buffer, 1 ml/kg). Standard diet was obtained from Animal Experimental Central of Shandong University (Jinan, China), and high-fat diet was purchased from Huafukang Company (Beijing, China). High-fat diet is composed of 1% cholesterol, 0.25% bile salt, 10% animal fat, 5% carbohydrate and standard diet. The model was successful on the basis of random blood glucose ≥ 16.7 mmol/L with other diabetic characters, such as hyperphagia, polydipsia, polyuria and weight loss.

### Metformin treatment

Metformin (Bristol-Myers Squibb) was dissolved in distilled water with the concentration of 100 mg/ml and then intragastrically administrated at the dosage of 500 mg/kg per day to rats in Group (D–/M+) throughout the experiment [34, 35]. The rats in Group (D+/M+) were given metformin by oral gavage for eight weeks after diabetes model induction. The other rats received daily intragastrically administrated with the same volume distilled water.

### Experimental design

Data on food intake and body weight were recorded once a week. Three days after injection of STZ, random blood glucose was measured by glucometer (Optium Xceed, USA) via the tail vein to monitor dynamic changes of all rats. At the end of the twelfth week, all the rats were fasted for 8 h before being sacrificed via cervical dislocation. Blood was collected from the retro-orbital plexus under ether anesthesia before they were sacrificed. Serum samples were obtained by centrifugation at 4°C (3000 r/min, 15 minutes) and stored in –80°C until analysis.

### Measurements of biomarkers estimations of fasting plasma glucose (FPG), fasting plasma insulin (FINS) and the homeostatic model assessment of insulin resistance (HOMA-IR) index

FPG was measured using glucometer (Optium Xceed, USA) by tail vein blood. FINS was measured using Elisa kit purchased from BlueGene Biotech (Shanghai, China; catalog no. E02I0004). HOMA-IR was made according to the following equation: HOMA-IR = FPG (mmol/L) × FINS(mIU/L)/22.5.

### Estimations of serum FT3, FT4 and TSH

At the end of the experimental period, fasting blood was collected for hormone analysis. Levels of serum FT3, FT4 and TSH were measured by Elisa using a commercial kit (BlueGene Biotech, Shanghai, China; FT3 kit, catalog no. E02F0247; FT4 kit, catalog no. E02F0251; TSH kit, catalog no. E02U0005).

### Statistical analysis

Data were presented as mean ± SD. Comparison of two group means was made with the *t*-test, a difference of *p* < 0.05 was considered statistically significant. All calculations were performed using SPSS 20.0 software (SPSS Inc, Chicago, IL, USA).
